# Minimally Invasive Plate Osteosynthesis with Mini-Open Technique and Supraclavicular Nerve Preservation Reduces Postoperative Numbness in Acute Displaced Midshaft Clavicle Fracture

**DOI:** 10.3390/medicina60101669

**Published:** 2024-10-11

**Authors:** Li-Tzu Liu, Jian-Chih Chen, Tsung-Cheng Yang, Hsiang-Jui Tseng, Shen-Ho Yen, Cheng-Chang Lu, Chung-Hwan Chen, Wan-Rong Chung, Ying-Chun Wang

**Affiliations:** 1 Department of Orthopedics, Kaohsiung Medical University Hospital, Kaohsiung Medical University, Kaohsiung 807, Taiwan; jessliu912@gmail.com (L.-T.L.); d830191@yahoo.com.tw (J.-C.C.); jero80723@gmail.com (H.-J.T.); cclu0880330@gmail.com (C.-C.L.); hwan@kmu.edu.tw (C.-H.C.); 2 Department of Orthopedics, College of Medicine, Kaohsiung Medical University, Kaohsiung 807, Taiwan; 3 Advanced Medical Devices and Composites Laboratory, Department of Fiber and Composite Materials, Feng Chia University, Taichung 407, Taiwan; 4 Department of Orthopedics, Kaohsiung Municipal Ta-Tung Hospital, Kaohsiung Medical University, Kaohsiung 801, Taiwan; bigricebug0614@gmail.com (T.-C.Y.); tennysonyen@gmail.com (S.-H.Y.); 5 Department of Orthopedics, Kaohsiung Municipal Siaogang Hospital, Kaohsiung Medical University, Kaohsiung 812, Taiwan; 6 Orthopedic Research Center, Kaohsiung Medical University, Kaohsiung 807, Taiwan; 7 Regeneration Medicine and Cell Therapy Research Center, Kaohsiung Medical University, Kaohsiung 807, Taiwan; 8 Department of Anesthesiology, Yuan’s General Hospital, Kaohsiung 802, Taiwan; wrchung1228@gmail.com

**Keywords:** clavicle, fracture, supraclavicular nerve, minimally invasive plate osteosynthesis

## Abstract

***Background:*** Minimally invasive plate osteosynthesis (MIPO) for clavicular shaft fracture yields favorable functional outcomes and results in less surgery-related soft tissue injury than other techniques. Anterior chest and shoulder skin numbness, a common complication after open reduction and plate fixation, is related to injury to the supraclavicular nerves. We propose MIPO combined with a mini-open approach without fluoroscopy for nerve preservation to minimize the risk of postoperative numbness compared with traditional open plating without nerve preservation. ***Methods:*** A total of 59 patients were retrospectively identified, with a follow-up period of 6 months. Thirty-two patients underwent MIPO with mini-open and nerve preservation technique (MIPO group), and 27 patients underwent traditional open plating without nerve preservation (open group). Constant–Murley shoulder outcome score, operation time, wound length, skin numbness, and number of implant removals were compared between the groups. ***Results:*** The MIPO group had significantly lower rates of anterior chest and shoulder skin numbness than the open group (MIPO: 12.5% vs. open: 55.6%; *p* < 0.001). Operation time was significantly longer in the MIPO group than in the open group (MIPO: 109.38 ± 18.83 vs. open: 81.48 ± 18.85; *p* < 0.001). Wound length was significantly shorter in the MIPO group than in the open group (MIPO: 4.73 ± 0.79 vs. open: 9.76 ± 1.64; *p* < 0.001). Both groups had similarly excellent Constant–Murley shoulder scores. There were significantly fewer implant removals in the MIPO group than in the open group (MIPO: 6.3% vs. open: 25.9%; *p* = 0.036). Neither group experienced any infection, implant failure, or nonunion. ***Conclusions:*** Our technique combining MIPO with the mini-open approach and supraclavicular nerve preservation yields a lower incidence of skin numbness than traditional open plating without nerve preservation.

## 1. Introduction

Clavicle fracture is a common traumatic injury around the shoulder. The middle third of the clavicular shaft is the most common fracture site. Conservative treatment was applied traditionally for displaced midshaft clavicle fractures. Studies have reported higher rates of malunion, nonunion, and poor functional outcomes for displaced midshaft clavicle fractures in adults after conservative treatment compared to surgery. In general, clavicle shortening of more than 2 cm (without cortical contact between the proximal and distal fragments) is commonly accepted as a criterion for surgical intervention in displaced midshaft clavicle fractures [1,2]. Open reduction and internal fixation (ORIF) has become the main treatment for displaced midshaft clavicle fracture, offering rapid functional recovery and lower complication rates than conservative treatment [3,4]. Various operative techniques for clavicle fracture fixation have been described, including titanium elastic nail [5], Knowles pin (K-pin) [6], and plate osteosynthesis fixation. The locking compression plate is a widely used implant for midclavicular fracture, offering rigid fixation and antirotation stability [7].

Anterior chest and shoulder skin numbness, a common complication after open reduction and plate fixation, is related to injury to the supraclavicular nerves. Studies have reported that sacrificing supraclavicular nerve branches can result in sensory deficits and even pain, with the incidence of postoperative numbness being as high as 55–86% in such cases [8,9]. The supraclavicular nerves are cutaneous and originate from the superficial cervical plexus at C3 and C4, branching into two to three groups. However, the course of the nerve branches around the clavicle is variable, hampering the identification of these nerves intraoperatively [10]. Because damage to the supraclavicular nerves leads to numbness and paresthesia of the chest and shoulder, identification and preservation of the nerves during surgical approach to the clavicle should be prioritized. Preserving the supraclavicular nerves during surgery can therefore reduce postoperative anterior chest wall numbness [11]. Minimally invasive plate osteosynthesis (MIPO) for clavicular shaft fracture yields favorable functional outcomes and results in less surgery-related soft tissue injury than traditional open plating [12]. Moreover, supraclavicular nerve injury after midshaft clavicle fracture surgery is less common in MIPO than in traditional open plating [13].

In this paper, we propose a mini-open technique for nerve preservation to minimize the risk of postoperative nerve numbness. To highlight the efficacy of our technique, we compared anterior chest and shoulder skin numbness and clinical outcomes in patients with midshaft clavicle fracture who underwent ORIF with plate fixation using different approaches for supraclavicular nerve preservation. To the best of our knowledge, this is the first report on MIPO combined with a mini-open approach without fluoroscopy for supraclavicular nerve preservation to reduce postoperative skin numbness in acute displaced midshaft clavicular fracture.

## 2. Materials and Methods

### 2.1. Patient Selection

We conducted a retrospective cohort study of patients with displaced 15.2A and 15.2B clavicle fractures (AO/OTA classification) who were treated in our institution from January 2016 to April 2021. The inclusion criteria were (1) having an acute displaced midshaft clavicle fracture (no cortical contact between the proximal and distal fragments on radiography or >2 cm of shortening), (2) being aged 18–85 years, and (3) being able to attend regular follow-up and provide complete information during the assessment. Patients with (1) pathologic fracture, (2) previous ipsilateral clavicle fracture history, or (3) open fracture or neurovascular-associated injuries were excluded. This study was conducted in accordance with the Declaration of Helsinki, and the protocol was approved by the Institutional Review Board of Kaohsiung Medical University Hospital, Kaohsiung, Taiwan (IRB NO: KMUHIRB-E(I)-20220229, Date of Approval: 9 November 2022).

We retrospectively identified 59 patients, with a follow-up period of 6 months. In total, 32 and 27 patients underwent MIPO with the mini-open and nerve preservation technique (MIPO group) and traditional surgery involving open plating without nerve preservation (open group), respectively. Constant–Murley shoulder outcome score, operation time, wound length, skin numbness, and number of implant removals were compared between the groups. We documented the following complications: infection, implant failure, and nonunion of the fracture.

### 2.2. Procedure

All surgical procedures were performed by a single senior orthopedic surgeon at our institution (Y.-C.W.). The surgical procedure in the two groups differed only in terms of skin incision, superficial dissection, and nerve preservation. All patients received the same anatomical clavicular plate (Synthes, Oberdorf, Switzerland) for fracture fixation. All patients underwent the operation in the beach chair position with the affected arm in a mobile position. Under general anesthesia, the entire shoulder was prepped and draped in a sterile manner. Prophylactic antibiotics were administered before skin incision.

### 2.3. Open Plating without Nerve Preservation Group (Open Group)

A transverse incision was made along the superior aspect of the clavicle. The incision length depended on the estimated plate length, which varies according to the fracture pattern. The surgeon dissected and penetrated through the muscle layer to expose the fracture site without identifying the supraclavicular nerves intraoperatively. Reduction was performed with minimal periosteal stripping. After temporary K-pin fixation of wedge fragments, anatomical reduction of the fracture site was conducted using interfragmentary screw fixation, if necessary. The operative wound was irrigated thoroughly with normal saline and closed in layers.

### 2.4. MIPO with Mini-Open and Nerve Preservation (MIPO Group)

**Figure 1:**
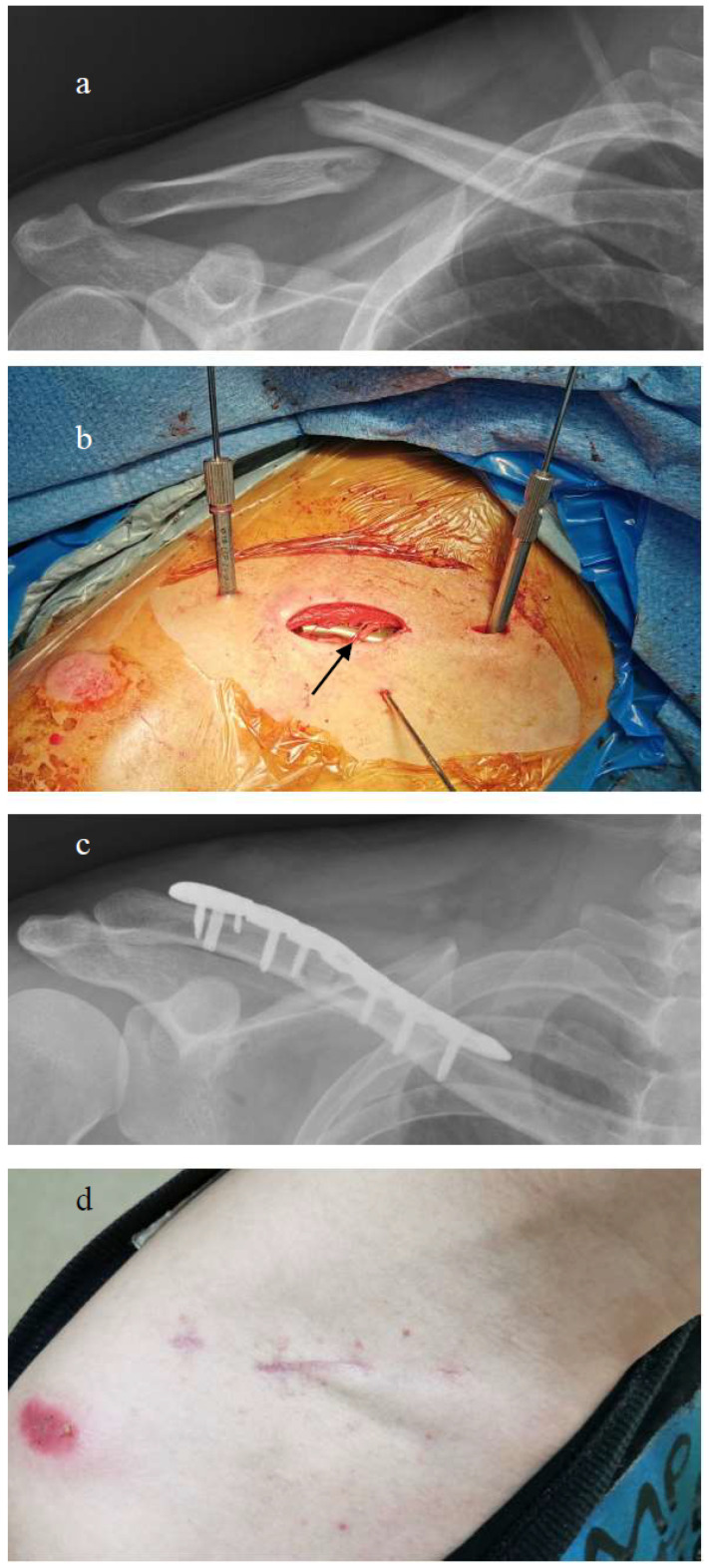
(**a**) Displaced midshaft clavicle fracture. (**b**) MIPO technique with mini-open approach and supraclavicular nerve preservation. Temporary K-wire was applied. Black arrow indicates preserved supraclavicular nerve. (**c**) Plate fixation of fracture. (**d**) Favorable wound condition at 1-month follow-up.

**Figure 2:**
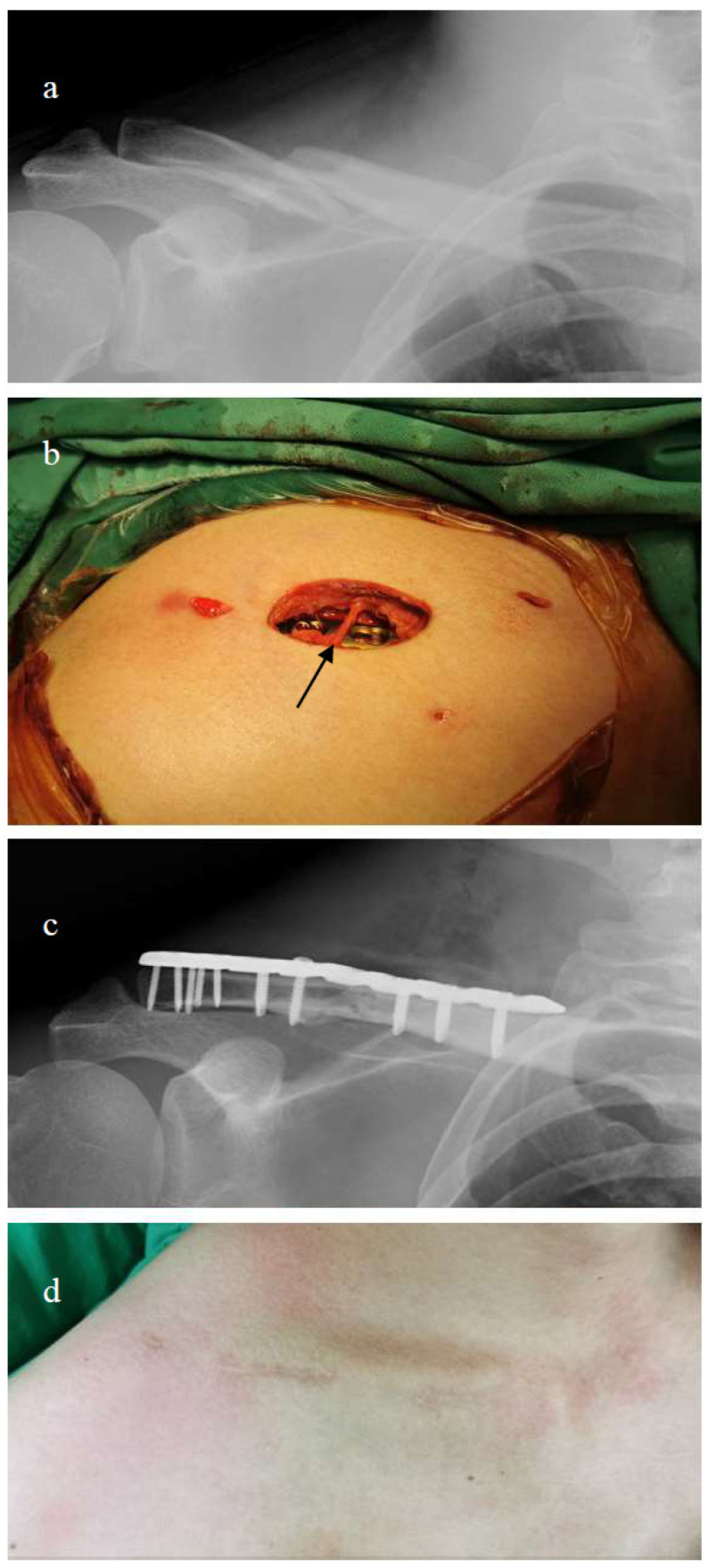
(**a**) Displaced midshaft clavicle fracture. (**b**) MIPO technique with mini-open approach and supraclavicular nerve preservation. Black arrow indicates preserved supraclavicular nerve. (**c**) Plate fixation of fracture. (**d**) Favorable wound condition at 1-month follow-up.

A small transverse skin incision approximately 2 cm above the fracture zone was made for open reduction. The surgeon dissected the superficial layer carefully and identified and protected the supraclavicular nerves during surgery while penetrating through the muscle layer to expose the fracture site. The incision length corresponded closely to that of the fracture zone to facilitate plate insertion for mini-open osteosynthesis. After temporary K-pin fixation of wedge fragments, anatomical reduction of the fracture site was conducted using interfragmentary screw fixation. The anatomical clavicular plate was subsequently inserted through the small skin incision and superiorly centered onto the clavicular shaft. The plate was adjusted medially and laterally to be placed at the fracture site. Because the distance between the clavicle and the skin is very thin, the anterior and posterior positions of the locking plate were palpated manually along the shaft of the clavicle. Additional small incisions (~1 cm) were made medially and laterally to the previous incision to drill the locking holes. The surgeon first fixed the plate with temporary K-pins and confirmed that the K-pins had penetrated through two layers of cortex. After confirming that the locking plate was centered on the bone, the surgeon applied the final locking screws through the drill holes under the small incision medially and laterally. The procedure was performed without fluoroscopic assistance. The operative wound was irrigated thoroughly with normal saline and closed in layers (Figure 1 and Figure 2).

### 2.5. Postoperative Follow-Up

The postoperative protocol was the same in both groups. The injured shoulder was protected with a shoulder sling for 2–4 weeks postoperatively, depending on the degree of fracture comminution. All patients underwent monthly follow-ups and radiographic evaluation until bone union was achieved; at the 6-month follow-up, the Constant–Murley shoulder outcome scores of patients were assessed. During the follow-up, the time of fracture union ranged between three and six months.

At the 6-month follow-up, we employed two methods for evaluating anterior chest and shoulder skin numbness at our outpatient department: (1) We asked the patients whether they experienced numbness over the anterior chest or shoulder area during the follow-up period. (2) The surgeon palpated gently over the anterior chest or shoulder area and asked whether the patient perceived any numbness.

The medical charts and radiographs of both groups were reviewed. We documented operation time, wound length, skin numbness, implant removal, and complications, namely infection, implant failure, and nonunion. Fracture healing was defined both clinically (no pain, no tenderness, ability to perform activities, and range of motion) and radiographically (trabeculae connecting both major fragments).

### 2.6. Statistical Analysis

All statistical analyses were performed using SPSS 20.0 (IBM, Armonk, NY, USA). We analyzed continuous data with equal variances using independent-sample *t* tests. For categorical data, we used the chi-square test. *p* < 0.05 was considered significant. A total sample size of 32 patients was determined using G*Power, version 3.1.9.7. The software settings are configured as follows: Test Family: chi-squared tests, Statistical test: Goodness-of-fit tests: Contingency tables, and Type of power analysis: A priori: Compute required sample size given alpha, power, and effect size. Then set effect size of 0.5, alpha level of 0.05, power of 0.80, and Df of 1.

## 3. Results

A total of 59 patients with midshaft clavicle fracture met our inclusion criteria. The average age was 42.00 ± 18.08 (range: 16–71) years in the MIPO group (17 men and 15 women). The average age was 49.89 ± 15.54 (range: 27–81) years in the open group (20 men and 7 women). The groups did not differ significantly in terms of age, sex, or fracture side (Table 1).

The operation time was significantly longer in the MIPO group than in the open group (MIPO: 109.38 ± 18.83 vs. open: 81.48 ± 18.85; *p* < 0.001). The wound length was significantly shorter in the MIPO group than in the open group (MIPO: 4.73 ± 0.79 vs. open: 9.76 ± 1.64; *p* < 0.001). The patients in the two groups had similarly excellent self-reported outcomes in terms of Constant–Murley shoulder scores (MIPO: 95.25 ± 4.45 vs. open: 93.33 ± 4.87; *p* = 0.120; Table 2).

The MIPO group had significantly lower rates of anterior chest and shoulder skin numbness than the open group. Four patients (12.5%) in the MIPO group reported skin numbness compared with fifteen patients (55.6%) in the open group (*p* < 0.001). Moreover, four patients in the MIPO group and twelve patients in the open group experienced skin numbness under gentle palpation by the surgeon at the 6-month follow-up, but they were not aware of any numbness in the preceding 6 months. Three patients in the open group had self-reported numbness after surgery, whereas no patient in the MIPO group reported this sensation. Two patients in the MIPO group and seven patients in the open group underwent implant removal surgery (6.3% vs. 25.9%, *p* = 0.036). The patients in the two groups did not experience any infection, implant failure, or non/mal union (Table 3).

## 4. Discussion

Our new technique combining MIPO with the mini-open approach and nerve preservation yielded a lower rate of skin numbness than the traditional open plating approach without nerve preservation over 6-month follow-up. Moreover, our proposed technique had similarly favorable functional outcomes to the traditional open plating approach. Although the new MIPO approach had a longer operation time than the open plating method, it resulted in smaller wounds and a lower rate of implant removal. To the best of our knowledge, our study is the first to use MIPO combined with a mini-open approach without fluoroscopy for supraclavicular nerve preservation to reduce postoperative skin numbness in acute displaced midshaft clavicular fracture.

Our method contrasts with the traditional MIPO approach by employing a mini-open technique that eliminates the need for fluoroscopy, thereby avoiding radiation exposure. At the same time, we retain the advantages of MIPO, such as aesthetically pleasing wounds and reduced nerve damage. Traditional open plating can also use nerve preservation techniques, and past research has confirmed that it can effectively reduce the risk of numbness [11]. However, it still does not address the issue of wound aesthetics. Therefore, our method combines the benefits of both approaches. Our data also demonstrate that it can effectively reduce wound size and decrease the likelihood of skin numbness.

Although numbness appears not to have an adverse effect on shoulder function, some patients may perceive numbness as adversely affecting their shoulder function [14,15,16]. Sacrificing supraclavicular nerve branches may result in sensory deficits and even pain [8,9]. Preserving these nerves during surgery can therefore reduce postoperative skin numbness [11]. Thus, identifying and preserving the nerves, whether using MIPO or open plating techniques during clavicle surgery, is paramount.

Because of the absence of a consensus on how to assess anterior chest and shoulder skin numbness, the incidence of postoperative supraclavicular nerve injury reported in the literature varies widely; for example, the rate of numbness after plate fixation can be as low as 10–29% or as high as 55–89% [8,9,11]. Moreover, the presence of numerous nerve branches and the variability in nerve injury depending on the approach and wound size complicate the evaluation of numbness. Inconsistent evaluation methods can result in an underestimation of the effect of postoperative cutaneous numbness on the patient after plate fixation for clavicle fracture. In our study, we adopted two methods for assessing postoperative numbness: self-reported numbness and numbness under palpation by a physician. More patients reported skin numbness while we palpated the skin rather than self-reporting this sensation over the follow-up period. We believe that self-reported numbness following surgery is more serious than numbness experienced only when palpated in a clinical setting. In the MIPO group, no patient self-reported numbness, and only four of them experienced numbness during palpation. In the open group, three of the fifteen patients self-reported numbness. Our technique thus reduces not only the rate but also the degree of numbness.

Beirer et al. analyzed a minimally invasive technique for clavicle fracture repair and found no differences between the MIPO and open methods regarding operation time [17]. However, in our study, the operation time in the MIPO group was longer than that in the open group. This difference can be attributed to more time being required for identifying and protecting the supraclavicular nerves during the operation despite the skin incision in the MIPO technique being smaller. In addition, because fluoroscopy was not used during surgery, more time was spent palpating the position of the plate relative to the clavicle to confirm correct placement. Even with the use of the mini-open technique, both groups underwent open reduction, allowing clear visualization of the fracture site. As a result, both groups achieved good reduction quality without experiencing malreduction.

Some researchers have also examined the impact of the skin incision type on postoperative skin numbness. Wang et al. reported that vertical incisions for the plate fixation of clavicle shaft fracture resulted in less postoperative numbness than horizontal incisions [16]. Shukla et al. found no difference in postoperative numbness between necklace and longitudinal incisions; however, patients in the necklace incision group were more satisfied with their scar appearance [18]. Li et al. used oblique incision to address concerns related to iatrogenic supraclavicular nerve injury and cosmetic appearance after plate fixation for clavicle fracture. They found that oblique incision had several advantages over transverse incision—smaller surgical wound, less injury to the supraclavicular nerves, and higher patient satisfaction—with equivalent effects on shoulder function recovery [19]. However, those methods are not widely used because they result in poorer exposure and reduction of the fracture than conventional incisions.

Researchers have observed favorable clinical outcomes when using the MIPO technique for clavicular shaft fracture repair [9,12,17,20,21,22]. Additionally, supraclavicular nerve injury after midshaft clavicle fracture surgery is less common in MIPO than in ORIF [13]. A systematic review and meta-analysis found no differences in functional outcomes and fracture healing between the MIPO and open plating techniques, a result consistent with ours [23]. However, because of the difficulty in applying a locking plate to the curved anatomy of the clavicle, using the MIPO technique for displaced midshaft clavicular fracture repair remains more challenging than the traditional open plating approach. Careful exposure and delamination of the supraclavicular nerves with a small incision during surgery can protect the nerves, resulting in satisfactory postoperative outcomes. In conclusion, our technique is safe and effective in reducing anterior chest and shoulder skin numbness after operative fixation of clavicle fracture. Our method facilitates the anatomic reduction of the fracture with a small skin incision and yields favorable functional outcomes.

Our study has some limitations. First, the quality of the data is limited by the inherent constraints of a retrospective study and the small number of patients, which may have introduced some bias. Additionally, we were unable to apply strict patient selection criteria during the experiment. Second, the postoperative follow-up period was relatively short, so we could not assess changes in numbness beyond 6 months. Third, we only measured subjective skin numbness. For measuring objective cutaneous hypoesthesia, further neurophysiological examinations with special instruments would have been necessary. Fourth, we only collected data on fracture patterns 15.2A and 15.2B. For other clavicle fracture patterns, we cannot determine whether this surgical method can be applied. Future prospective studies with longer follow-up could clarify the difference and efficacy of the two techniques and avoid the potential bias inherent in a retrospective methodology.

## 5. Conclusions

Since anterior chest and shoulder skin numbness is a common complication following open reduction and plate fixation for clavicle fractures, prioritizing strategies to prevent this issue is essential. Our technique combining MIPO with the mini-open approach and supraclavicular nerve preservation yields a lower incidence of skin numbness than traditional open plating without nerve preservation. Although the operation time may be longer, for physicians seeking to reduce postoperative skin numbness, our surgical technique offers a possible direction for clavicle surgery.

## Figures and Tables

**Table 1:** Main characteristics of the patients in each group.

	MIPO (n = 32)	Open (n = 27)	*p*-Value
**Age (year)**	42.00 ± 18.08	49.89 ± 15.54	0.080
**Gender**			
Male, (n)	17	20	0.097
Female, (n)	15	7	
**Affected Side**			
Right	13	10	0.778
Left	19	17	

**Table 2:** Operation time, wound length, and functional outcome in the two groups.

	MIPO (n = 32)	Open (n = 27)	*p*-Value
**Skin Numbness**	4 (12.5%)	15 (55.6%)	<0.001 *
Numbness under palpation	4	12	
Self-aware of numbness	0	3	
**Implant Removal**	2 (6.3%)	7 (25.9%)	0.036 *
**Complications**			
Infection	0	0	
Implant Failure	0	0	
Non/mal Union	0	0	

* *p*-value < 0.05.

**Table 3:** Skin numbness, implant removal, and complications of the included patients in the two treatment groups.

	MIPO (n = 32)	Open (n = 27)	*p*-Value
**Operation Time (minutes)**	109.38 ± 18.83	81.48 ± 18.85	<0.001 *
**Wound Length (cm)**	4.73 ± 0.79	9.76 ± 1.64	<0.001 *
**Constant Score**	95.25 ± 4.45	93.33 ± 4.87	0.120

* *p*-value < 0.05.

## Data Availability

The data presented in this study are available upon request from the corresponding author. The data are not publicly available because of confidentiality issues.
